# A simple and reliable approach for the fabrication of nanoporous silver patterns for surface-enhanced Raman spectroscopy applications

**DOI:** 10.1038/s41598-021-01727-z

**Published:** 2021-11-16

**Authors:** Angela Capaccio, Antonio Sasso, Giulia Rusciano

**Affiliations:** 1 Department of Physics ‘E. Pancini’, University of Naples Federico II, Via Cintia, 80126 Naples, Italy; 2 National Institute of Optics (INO)-National Research Council (CNR), Via Campi Flegrei 34, 80078 Pozzuoli, NA Italy

**Keywords:** Nanoparticles, Nanoscale materials, Optical spectroscopy

## Abstract

The fabrication of plasmonic nanostructures with a reliable, low cost and easy approach has become a crucial and urgent challenge in many fields, including surface-enhanced Raman spectroscopy (SERS) based applications. In this frame, nanoporous metal films are quite attractive, due to their intrinsic large surface area and high density of metal nanogaps, acting as hot-spots for Raman signal enhancement. In this paper, we report a detailed study on the fabrication of nanoporous silver-based SERS substrates, obtained by the application of two successive treatments with an Inductively Coupled Plasma (ICP) system, using synthetic air and *Ar* as feeding gases. The obtained substrates exhibit a quite broad plasmonic response, covering the Vis–NIR range, and an enhancement factor reaching 6.5 $\times\, 10^7$, estimated by using *4-mercaptobenzoic acid* (4-MBA) as probe molecule at 532 nm. Moreover, the substrates exhibit a quite good spatial reproducibility on a centimeter scale, which assures a good signal stability for analytical measurements. Globally, the developed protocol is easy and cost effective, potentially usable also for mass production thanks to the remarkable inter-batches reproducibility. As such, it holds promise for its use in SERS-based sensing platforms for sensitive detection of targets molecules.

## Introduction

Sensitive detection of analytes is of paramount importance in a broad range of scientific and technological fields, such as biomedicine, cells imaging, molecular diagnostics, drug development and pollutants detection^1–5^. Surface-enhanced Raman spectroscopy (SERS)^6–8^ has recently gained a high and increasing attention to this purpose, thanks to its remarkable sensitivity (up to single molecule) and chemical specificity, due to the well-known “fingerprint” character of Raman spectroscopy. The outstanding SERS sensitivity relies on the excitation of localized surface plasmon resonances in proper metal (mainly silver and gold) nanostructures, which provide a huge enhancement of the electromagnetic field felt by molecules located near the so-called “hot spots”^9^. The fundamental metric for SERS is the Enhancement Factor (EF), which quantifies the increase of signal intensity per molecule with respect to the spontaneous Raman case. In principle, EF up to 10$^{14}$ can be predicted, although EF $\sim$ 10$^{5}$–10$^{7}$ are more realistically achieved. Apart from this parameter, SERS substrate uniformity is equally important for analytical applications of SERS, as it guarantees the independence of the acquired spectrum from the observed substrate site. Finally, it is also crucial to control the variability among SERS substrates from different production batches.

To improve SERS performances, numerous strategies have been reported, devoted to increase both EF through the optimization of the metal nanostructure architecture (in terms of size, shape and interparticles distance) and substrate uniformity, mainly by tacking advantage of bottom-up self-assembling approaches^10–13^. Alternatively, highly homogeneous and uniform substrates can be achieved by physical nanopatterning techniques based on electron-beam lithography (EBL) or on nano-imprinting lithography^14,15^. However, these approaches are quite expensive and the realization of large area substrates is time consuming.

In this frame, the production of metal nanoporous films based on chemical-physical methods are gaining ground due to the high plasmonic performances of such structures and the relatively simple set-ups required^16–21^. Nanoporous materials are characterized by an intriguing network of interconnected structures with open nanopores giving rise to a large specific surface area. Numerous studies have been reported regarding the use of nanoporous materials for electrocatalytic applications, photovoltaic devices and biosensing platforms, including SERS-based sensors^22–25^. In this regard, the main advantages of nanoporous SERS substrates concern on (i) the large internal surface available for molecules anchoring, which obviously extends the use of SERS to higher concentrations, and (ii) the high density of hot-spots formed in the nanopores, which assures an adequate enhancement for analyte detection. High enhancing nanoporous SERS substrates can be easily obtained from salt induced random aggregations of colloidal nanoparticles, which, however, pay the price in terms of a quite poor spatial uniformity and inter-batches reproducibility. More reliable substrates are provided by electrochemical dealloying, selective etching in gold-silver alloy, vapor-phase dealloying and sacrificial template approaches^26^.

In this study, we report on an easy approach to produce large-area, Ag-based nanoporous SERS substrates. In particular, starting from a relative flat Ag-film deposited by magnetron-sputtering on a glass coverslip, a significant surface roughening is obtained by an Inductively-Coupled-Plasma (ICP) treatment, using synthetic air as feeding gas. This procedure, previously demonstrated to induce roughening on metallized AFM tips^27^, gives rise to a *coral-like* nanopattern, mainly composed of AgO. Therefore, a further ICP treatment based on a Ar plasma discharge reduces AgO, restoring the pristine metallic silver. Herein, we present a systematical characterization, from both morphological and plasmonic point of view, of the silver nano-pattern obtained by changing several key-parameters of the whole production process. In particular, by using *4-mercaptobenzoic acid* (4-MBA) as probe molecule, we demonstrate that an EF up to 6.5 $\times\, 10^7$ can be obtained. Moreover, silver nanopatterns present a certain degree of order, revealed by Fast Fourier Analysis. This feature can be reasonably attributed to the spinodal dewetting (SD), a Solid-State Dewetting (SSD) mechanism^28^ observed in metastable thin films^29^, that could play an important role for the formation of the observed nanopatterns. The analysis shown herein also reveals a quite good substrate spatial uniformity and inter-batch variability, which are fundamental for analytical measurements. Furthermore, the substrate aging and reusability were investigated, which proves that a proper Ar-based treatment is able to restore to a great extent the initial substrate performances. Finally, the substrate wettability and its evolution under substrate storage at room environment were also investigated. Also in this case, Ar-plasma is able to restore the initial substrate hydrophilicity, which is fundamental to assure a uniform distribution of molecules on the SERS substrate.

## Results

### SERS substrates production

Globally, the whole SERS substrate production process can be divided into three steps:

- Deposition of a Cr/Au/Ag multilayered film.
- Ag-layer roughening by air-based plasma.
- Silver oxide reduction by Ar-based plasma.

A similar procedure has been previously reported by our group for the production of active plasmonic probes for TERS applications, starting from commercial silicon-based AFM tips^27^. In the present case, instead, the porous silver nanotexture was created on commercial glass coverslips, in order to produce high-enhancing SERS substrates. In this respect, all the process phases were optimized in order to obtain a homogeneous nanotexture over a large area rather than on the nanosized AFM tip. This was accomplished through a parametric characterization on the substrate performances as result of the variation of several key parameters, such as the Ag-layer thickness ($d_{Ag}$) and the exposure time to both the air-based plasma ($\tau _{air}$) and the Ar-based plasma ($\tau _{Ar}$), as described below.

#### Deposition of a Cr/Au/Ag multilayered film

Initially, a 3 nm-Cr/10 nm-Au bilayer was sputtered on a clean 15 mm $\times$ 15 mm glass coverslip. This step is essential in order to improve the adhesion of the overlying Ag layer during the successive plasma treatment (see below). At this purpose, we employed a magnetron sputtering system (Quorum, Q300TD), endowed with two independent sputtering heads, therefore allowing the sequential deposition of the two metal films without breaking the vacuum in the chamber. The thickness of the two metal films were monitored during the deposition through the use of two quartz microbalances. The sputtering rates were controlled by the sputtering currents, which were $\hbox {I}_{{Cr}}$ = 120 mA and $\hbox {I}_{{Au}}$ = 20 mA for Cr- and Au-layer deposition, respectively. Finally, an Ag-film was sputtered on the adhesion bilayer, at a sputtering current $\hbox {I}_{{Ag}}$ = 50 mA. The silver film thickness $d_{Ag}$ was optimized in the 10–200 nm range. In all the cases, the Ag-film appeared quite smooth on nanometric scale, with a roughness $\sim$ 0.8 nm, as revealed by AFM analysis (see Fig. S1 in the Supplementary Information).

#### Ag-layer roughening by air-based plasma

**Figure 1:**
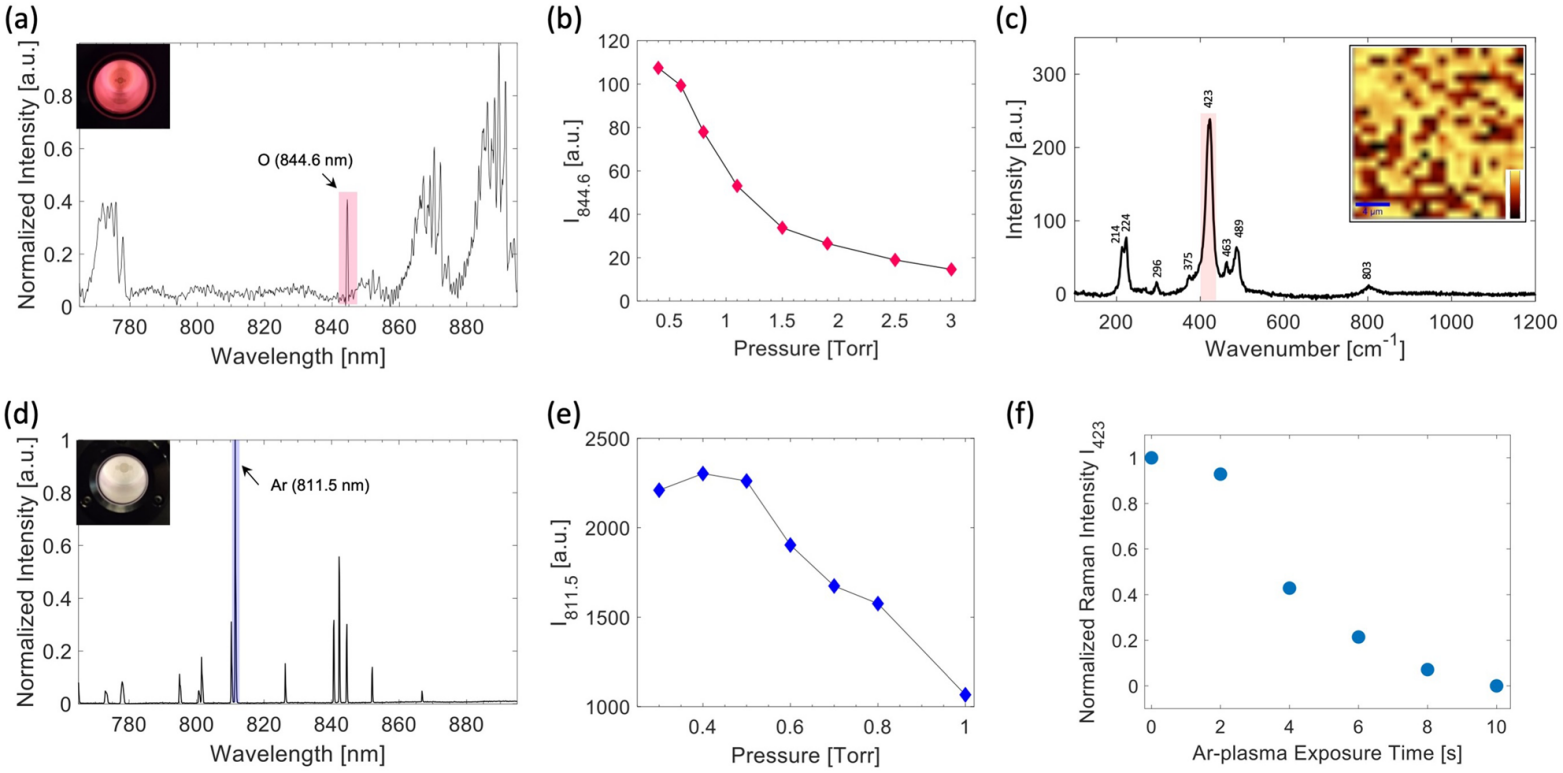
**(a)** Emission spectrum of the air-based plasma used to induce the silver layer nanostructuring, obtained at an air pressure $\hbox {P}_{{air}}$ = 0.4 Torr and a RF power $\hbox {P}_{{RF}}$ = 18 W. The peak highlighted by the pink bar is due to atomic oxygen and it was used for monitoring the formation of this species. The inset reports a picture of the plasma discharge, exhibiting a pink-orange color following atomic oxygen production optimization. **(b)** Intensity of the O peak at 844.6 nm *vs* $\hbox {P}_{{air}}$, obtained at $\hbox {P}_{{RF}}$ = 18 W. **(c)** Raman spectrum of a 30 nm Ag layer treated in air-plasma for 90 s; it was obtained by using a power of P = 70 $\upmu$W and an integration time of 100 s. The inset shows the Raman map corresponding to the integrated intensity of the peak at 423 cm$^{-1}$ (highlighted in the spectrum by the pink bar) acquired over a 20 $\times$ 20 $\upmu$m$^2$ region with a 1 $\upmu$m step. The Raman probe power and the integration time were 0.5 mW and 2 s, respectively. **(d)** Emission spectrum of the plasma, obtained by using Ar as feeding gas at a pressure $\hbox {P}_{{Ar}}$ = 0.4 Torr and $\hbox {P}_{{RF}}$ = 18 **W**. The inset shows a picture of such plasma. **(e)** Intensity of the Ar peak at 811.5 nm *vs* $\hbox {P}_{{Ar}}$, obtained at $\hbox {P}_{{RF}}$ = 18 W. **(f)** Evolution of the AgO peak at 423 cm$^{-1}$ at different $\tau _{Ar}$.

After that, the substrates were exposed to the RF air-based plasma in order to induce the nanostructuring of the Ag film. The plasma system is the Harrick Plasma PDC-32G-2 instrument, commonly used for surfaces cleaning and activation. As better discussed in the following, atomic oxygen created in the air-based plasma plays a key role in the morphological modification of the Ag surface. Therefore, in order to develop a robust and reproducible fabrication protocol, it was crucial to monitor the formation of this species in the plasma chamber. As a matter of fact, a rough indication for the efficiency in the production of this species is surely the plasma discharge color, which, in the best condition, is an intense pink-orange (see the inset of Fig. 1a). However, a more effective and quantitative process control was achieved by monitoring the plasma emission spectrum in the 765–895 nm region. At this purpose, we collected the fluorescence of the plasma glow discharge through an optical fiber and sent it to a IR-spectrometer for the spectral analysis. A typical emission spectrum acquired by this system is reported in Fig. 1a. It corresponds to the emission of the air-based plasma at an air pressure in the chamber $\hbox {P}_{{air}}$ = 0.4 Torr and a RF power $\hbox {P}_{{RF}}$ = 18 W. The spectrum, which is dominated by $\hbox {O}_2$ and $\hbox {N}_2$ molecular bands, also exhibits two narrow peaks, at 777.5 nm and 844.6 nm, assigned to atomic oxygen. In particular, the peak at $\sim$ 844.6 nm corresponds to the O($3p^3P \rightarrow 3s^3S$) atomic oxygen transition, so that it can serve as a quite useful observable to monitor the production of this species. Typically, the production of atomic oxygen in electrical discharges is enhanced when $\hbox {O}_2$ is in presence of a buffer gas (usually noble gases) with respect to the case of pure $\hbox {O}_2$^30^. In particular, the atomic oxygen is maximized at a particular value of the gas pressure, as a consequence of the equilibrium between the dissociation of $\hbox {O}_2$ molecules and the recombination of O atoms to re-form $\hbox {O}_2$. Following this approach, we first optimized O production as function of the stationary air pressure $\hbox {P}_{{air}}$ in the chamber, obtained by varying the feeding gas flux. Figure 1b reports the intensity $\hbox {I}_{844.6}$ of the previously mentioned O peak *vs* $\hbox {P}_{{air}}$. Clearly, the more efficient O production is obtained at the lower pressure value here analyzed, so that $\hbox {P}_{{air}}$ = 0.4 Torr was selected for SERS substrate production. A further pressure decrease is accompanied, in our system, to a flux instability, so that this option was discarded.

Once optimized $\hbox {P}_{{air}}$ in the plasma chamber, we analyzed the effect of the air-based plasma on the silver layer. In particular, we analyzed the substrate nanostructuring at different $\tau _{air}$, in the 20–250 s range, keeping $d_{Ag}$ = 30 nm. It turned out that a nanoporous *coral-like* morphology can been observed for $\tau _{air}$ ranging from 60 s to 180 s. At higher $\tau _{air}$ values the deposition layer starts to be exfoliated from the glass support, and the silver nano-pattern uniformity is definitely lost. A complete characterization of the substrate morphology at different $\tau _{air}$ values is reported in the next paragraph. On the other hand, at fixed $\tau _{air}$ = 90 s, the *coral-like* morphology has been observed in all the $d_{Ag}$ range (10–200 nm) explored in this study, although for $d_{Ag}$ = 10 nm the substrate uniformity, from a morphological point of view, was sometime lost.

Following the application of air-based plasma, the substrates were analyzed by Raman spectroscopy. The Fig. 1c reports the Raman spectrum obtained by using a Raman probe power P = 70 $\upmu$W and an integration time $\tau$ = 100 s for the substrate obtained with $d_{Ag}$ = 30 nm and $\tau _{air}$ = 90 s. The bands observed at 214, 224, 296, 375, 423, 463 and 489 cm$^{-1}$ can be ascribed to AgO, while there is no spectroscopic evidence of $\hbox {Ag}_2$O, usually appearing as a broad feature at $\sim$ 490 cm$^{-1}$^31^. Roughly, AgO is quite uniformly distributed on the whole substrate, as evident from the map in the inset of Fig. 1c, which reports the intensity of the AgO peak at 423 cm$^{-1}$. As it is well known, silver oxides are rather sensitive to photochemical reduction and re-oxidation^32^. As a consequence, prolonged exposition to relatively high laser power leads to a chemical change of the surface structure. This feature, which is important for the stability of our substrates, was monitored by spontaneous Raman spectroscopy. The obtained results are provided as Supplementary Information.

#### Silver oxide reduction by Ar-based plasma

The final step of our protocol consists in the exposure of the oxidized substrates to *Ar* plasma. This treatment is effective to reduce the Ag-oxide^33^ with no significant influence on the surface morphology. Also in this case, the plasma stability was monitored by looking at its emission spectrum in the near-IR region, which is reported in Fig. 1d. In this case, the plasma discharge was monitored by looking at the Ar $2p_9 \rightarrow 1s_5$ transition (811.5 nm) involving the metastable $1s_5$ level, which is populated by electronic collisions. Since metastable atoms constitute a reservoir of excited species which contribute to the formation of ions through electronic collisions^34,35^, the intensity $\hbox {I}_{811.5}$ of this band actually allows to estimate the production of Ar$^+$ *ions* ruling the reduction process. Figure 1e reports $\hbox {I}_{811.5}$ *vs* $\hbox {P}_{{Ar}}$. This analysis reveals that, also in this case, the production of ionized species is optimized in the low pressure region, so that the $\hbox {P}_{{Ar}}$ = 0.4 Torr value was selected. Finally, the surface chemistry was again investigated by Raman analysis as function of $\tau _{Ar}$. For such measurements, the laser power was limited to 0.3 mW, in order to avoid photo-induced effects. Figure 1f reports the intensity $\hbox {I}_{{423}}$ of the AgO band at 423 cm$^{-1}$ as function of the total exposure time to the Ar-plasma. Notably, after 10 s the AgO Raman band is no more visible, indicating a substantial decrease of AgO layer. Of course, due to the limited sensitivity of spontaneous Raman analysis, this cannot be intended as a complete reduction of AgO to metallic Ag. As a matter of facts, the best performances (from the plasmonic point of view) of our substrates were observed after an exposure time $\tau _{Ar}$ = 50 s, that therefore was chosen for the preparation protocol of our SERS substrates.

### Morphological characterization and insight on the nanopatterning mechanism

**Figure 2:**
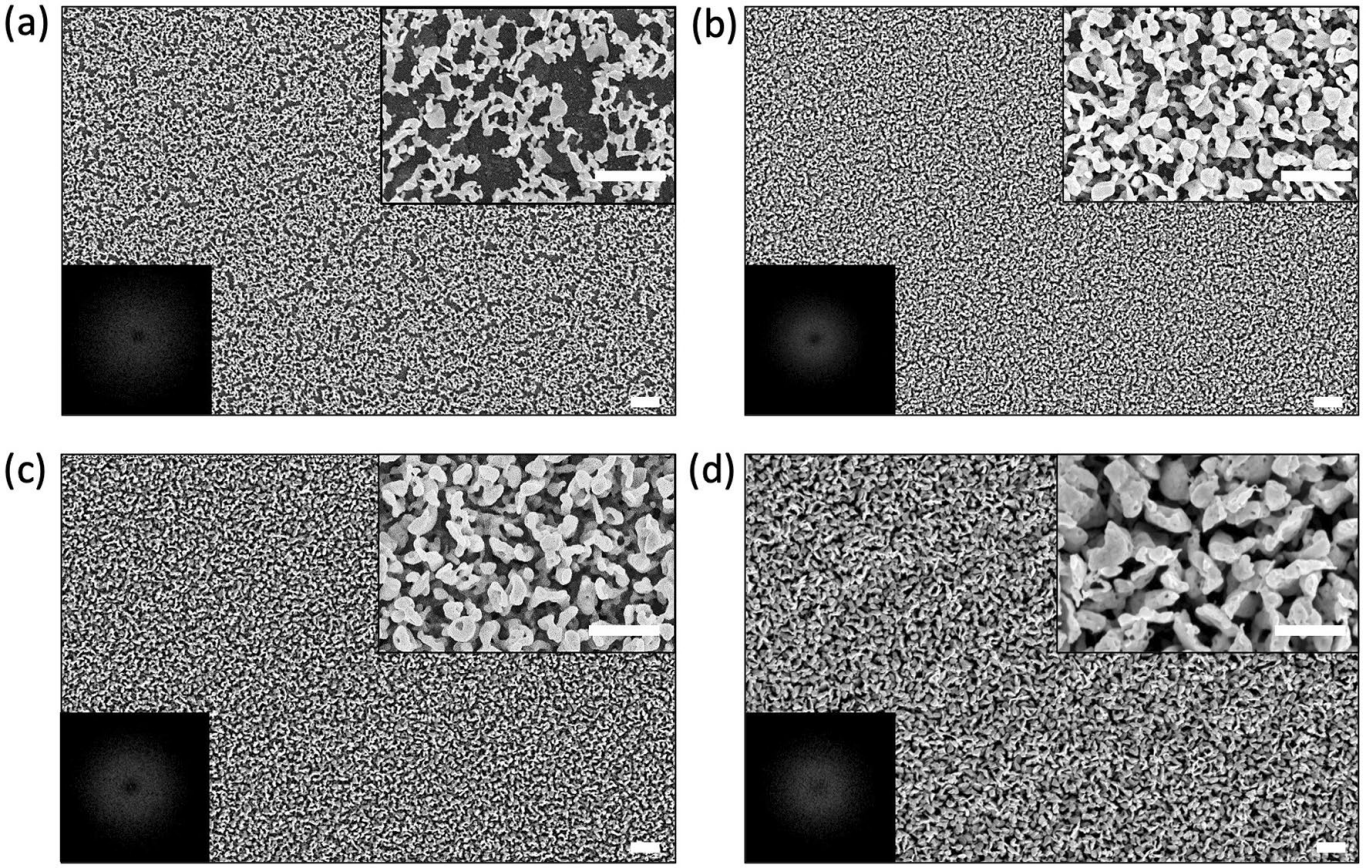
SEM images of SERS substrates covered by a **(a)** 10 nm, **(b)** 30 nm, **(c)** 50 nm and **(d)** 200 nm Ag layer and treated for 90 s with the air-based plasma and for 50 s with the Ar-based plasma (scale bar = 1 $\upmu$m). The insets in the upper right corners show the magnification of the SEM image (scale bar = 500 nm), whereas, the insets in the lower left corners show the PSD.

As mentioned above, the Ag-film thickness $d_{Ag}$ and the exposure time to air-based plasma $\tau _{air}$ are expected to play a role in Ag film nanostructuring. Therefore, the morphology of the SERS substrates was studied through a systematic variation of these two parameters. Figure 2a–d shows the SEM images of SERS substrates obtained by keeping constant $\tau _{air}$ to 90 s and changing $d_{Ag}$. Starting from such images, we analyzed the size of the irregularly shaped nanostructures by calculating $d_{EQPC}$, corresponding to diameter of a circle of equal projection area^36^. The obtained results are reported in Table 1. As it is possible to see, $d_{EQPC}$ tends to increase with $d_{Ag}$, as usually found in SSD process^28^. On the other, measurements from substrates obtained at a fixed $d_{Ag}$ by varying $\tau _{air}$, show that $d_{EQPC}$ remains roughly constant. Intriguingly, in all the cases, the observed nanopattern also presents a certain degree of order, as revealed by Fourier analysis. In particular, the PSD calculated for each image, shown in the insets of Fig. 2, reveal a well-defined ring-shape, that suggests an isotropy of our substrates and the existence of a long-range order with the characteristic wavelength $\lambda _c$. In particular, once the nanopattern formation has been triggered ($\tau _{air}$ $\ge$ 60 s), no obvious dependence of $\lambda _c$ from the process parameters has been observed.

**Table 1:** Surface parameters obtained by varying $d_{Ag}$ at fixed $\tau _{air}$ = 90 s (left part) and by varying $\tau _{air}$ at fixed $d_{Ag}$ = 30 nm (right part).

	Ag thickness (nm)				Exposure time (s)		
	($\tau _{air}$ = 90 s)				($d_{Ag}$ = 30 nm)		
	10	30	50	200	90	120	180
$\lambda _c$ (nm)*	$285 \pm 2$	$262 \pm 1$	$347 \pm 2$	$432 \pm 2$	$262 \pm 1$	$290 \pm 2$	$338 \pm 2$
$d_{EQPC}$ (nm)**	88 (64, 118)	109 (71, 141)	135 (92, 168)	168 (92, 219)	109 (71, 141)	111 (84, 131)	126 (81, 163)

*Errors on $\lambda _c$ result from the fitting with a Lorentzian function.

**Median (lower quartile, upper quartile).

Such feature provides us some insight on the mechanism ruling the formation of the observed *coral-like* nanopattern. As a matter of facts, multiple effects can take place during the ICP treatment.

i. As first, the oxidation process taking place in the RF plasma could contribute to the break-up of the silver surface. In fact, as the oxide layer grows, internal stresses due to mechanical or thermal excursions can induce the cracking of the formed oxide layer. As result, the pristine underlying silver exposed again to the air plasma repeats the process until the whole silver gets a complete oxidization, as described in Ref.^37^.
ii. Another mechanism that could play a role in the nanopattern formation is the Kirkendall effect^38^, a diffusional phenomenon that can occur at the interface between two different materials. In this case, the difference in the diffusion rates of the two constituents produces a disequilibrium of the material flow that is compensated by the inter-diffusion of vacancies. In some cases, the condensation of excess vacancies can result in the formation of voids, called Kirkendall voids, which globally give rise to a porous nanostructure. The Kirkendall effect is activated at high temperature (hundreds of degrees) so that it is not expected in a cold plasma discharge. However, as also suggested in Ref.^38^, the temperature could rise locally due to the exothermic nature of the chemical oxidation reaction of the silver and/or the conversion into heat of the kinetic energy carried by ions in the discharge impacting on the silver film.
iii. Also connected to the local temperature rise, SSD is surely an important co-factor in the silver layer nanopatterning, as previously reported^39,40^. In particular, SD, often occurring in very thin films, could trigger the formation of the *coral-like* structure. According to literature^29^, SD occurs in metastable films through the amplification of thermally-induced thickness perturbations. Notably, this mechanism leads to nanostructures which are spatially correlated, presenting a characteristic correlation length which increases with the square of film thickness^29^.

In such frame, is quite difficult to distinguish the contribution of the different mechanisms, which could be important in the different phases of nano-pattern formation. However, due to the presence of a characteristic correlation length associated to substrate morphology, we hypothesize that SD could certainly contribute to nanopattern formation. In particular, we speculate that SD occurs in the thin oxide layer formed during the early stages of the process. As a matter of fact, due to low thermal conductivity of oxide, it is reasonable to envisage a local rise in temperature of this film. As plasma treatment proceeds, temperature get stored in the oxide film, so that it can rise up, finally triggering film dewetting. Clearly, in order to confirm this scenario, it would be quite useful to measure the local silver oxide film temperature. Moreover, an accurate measurement of the oxide film thickness could be help in verifying the dependence of the correlation length from film thickness (expected for spinodal dewetting). Both items are not easy tasks, and they are out of the scope of the present investigation.

### Plasmonic characterization

#### Enhancement factor

**Figure 3:**
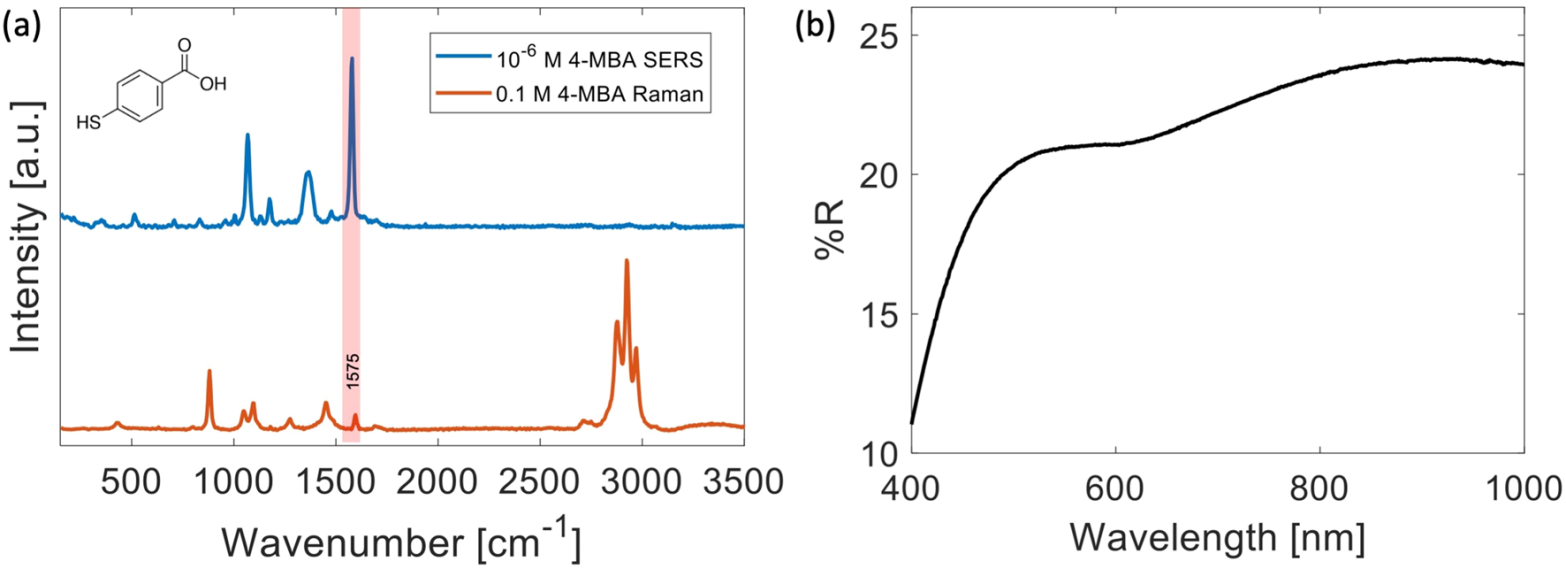
**(a)** Comparison between the spontaneous Raman and SERS spectrum of 4-MBA. The first signal was obtained at a 0.1 M concentration, while the Raman probe power and the integration time were $\hbox {P}_{{Raman}}$ = 15 mW and $\tau _{Raman}$ = 2 s, respectively. SERS spectrum is the average of 400 signals obtained in a raster scan of a 10 $\upmu$m $\times$ 10 $\upmu$m region. Each spectrum of the scan was acquired by using a probe power $\hbox {P}_{{SERS}}$ = 10 $\upmu$W and an integration time $\tau _{SERS}$ = 1 s. The pink bar highlights the spectral band used to estimate the EF. It is worth noticing that the 4-MBA peak selected for the EF estimation is free from interferences from the solvent. **(b)** Diffuse reflectance curve relative to the substrate obtained by using $d_{Ag}$ = 30 nm and $\tau _{air}$ = 90 s.

The enhancement factor of our SERS substrates was estimated by using 4-MBA as probe molecule. According to Ref.^41^, this molecular species appears ideally suited for modeling the plasmonic response of nanostructured materials. In particular, we analyzed substrates obtained starting from $d_{Ag}$ = 30 nm, corresponding to the smallest Ag film thickness giving rise to a reproducible *coral-like* nanopattern. Figure 3a reports both the Raman spectrum of 0.1 M 4-MBA ethanol solution (lower trace) and the SERS spectrum of a 10$^{-6}$ M 4-MBA solution adsorbed on the SERS substrate obtained with $\tau _{air}$ = 90 s (upper trace). In order to take into account the point-to-point signal fluctuations, the SERS spectrum was obtained from the average of 400 signals obtained from a raster scan in a 10 $\upmu$m $\times$ 10 $\upmu$m region. Notably, the SERS spectrum is consistent with that reported in previous papers^21,42^ and is slight different from the spontaneous Raman signal, as result of surface selection rules. The most prominent bands correspond to the ring breathing modes of 4-MBA, observed at $\sim$ 1070 and 1575 cm$^{-1}$. The relatively broad band around 1360 cm$^{-1}$ can be instead attributed to the symmetric stretch of the carboxylate group COO$^-$, which appears in the spontaneous Raman spectrum at $\sim$ 1410 cm$^{-1}$. As previously reported^19,43^, such shift can be attributed to COO-Ag interaction, and is particularly large ($\sim$ 50 cm$^{-1}$) as result of the nanoporous geometry of the substrates, which allows both the thiol and COO$^-$ group of 4-MBA to bind in a sandwich structure. In line with this conjecture, a smaller ($\sim$ 30 cm$^{-1}$) shift has been observed for more planar 2D SERS surfaces^43,44^. In order to estimate the EF, the intensity of the $\nu$(C–C) ring-breathing mode at 1575 cm$^{-1}$, clearly visible in both Raman and SERS spectrum, was considered. After a proper signal normalization with respect to the Raman probe power and integration time, EF was calculated according to the relation:

1 $$\begin{aligned} EF = \frac{I_{SERS}}{I_{Raman}} \times \frac{N_{Raman}}{N_{SERS}}, \end{aligned}$$

where $I_{SERS}$ and $I_{Raman}$ are the intensities of SERS and Raman bands generated by a number of scattering molecules $N_{SERS}$ and $N_{Raman}$, respectively. $N_{Raman}$ was estimated from the relation $N_{Raman}$ = [4-MBA]$_{Raman}\times V_{conf}\times$ $N_A$, being $N_A$ the Avogadro number. On the other hand, $N_{SERS}$ was estimated starting from the molecular surface density $\sigma _S$ (molecules adhered on SERS substrate divided by the apparent geometrical surface area) and the confocal area $A_{conf}$ probed by the Raman beam, according to the relation $N_{SERS}={\sigma _S}\times A_{conf}$. In our experimental conditions, we obtained $N_{SERS}$ = 1.18 $\times\, 10^4$ and $N_{Raman}$ = 1.38 $\times\, 10^8$, so that EF $\sim$ 6.6 $\times\, 10^7$.

The EF was also estimated by using adenine as probe molecule. This molecular species presents a high affinity to silver and is non-resonant for excitation at 532 nm. At this purpose, adenine molecules were dissolved in distilled water (pH 7) and both the spontaneous and the SERS spectra were acquired following a procedure similar to that used for 4-MBA. The outcomes of this analysis are provided as Supplementary Information. Briefly, a EF $\sim 0.8 \times\, 10^7$ has been estimated. It is worth noticing that, according to Ref.^45^,an increase of the measured EF (up to almost an order of magnitude) can be obtained by increasing the buffer pH value from 7 to 9.

As a final step, we also estimated the variability of the plasmonic performances of substrates obtained in different production batches. This was done by estimating the EF from substrates prepared in 5 independent protocol runs, using 4-MBA as probe molecule. This analysis provides EF = (6.5 ± 0.2) $\times$ 10$^7$, so that a $\sim$ 30% inter-batches variability can be estimated.

A similar investigation has been applied to evaluate the performances of SERS substrates with $d_{Ag}$ = 30 nm and prepared by using different $\tau _{air}$ in the 60-180 s range. The results are summarized in Table 2.

**Table 2:** EF values obtained with $d_{Ag}$ = 30 nm and different $\tau _{air}$.

$\tau _{air}$	EF
60	($4.5 \pm 0.2$) $\times$ 10$^5$
90	($6.5 \pm 0.2$) $\times$ 10$^7$
120	($7.3 \pm 0.3$) $\times$ 10$^6$
150	($8.4 \pm 0.4$) $\times$ 10$^6$
180	($2.0 \pm 0.3$) $\times$ 10$^5$

The standard deviations *correspond* to repeated measurements on substrates from 5 different production batches.

As it is possible to note, the higher EF is obtained for $\tau$ = 90 s, while a decrease is observed for larger exposure times. According to this outcome, the substrate obtained with $\tau$ = 90 s exhibiting the highest EF was selected for all the successive characterizations.

#### Diffuse reflectance

In order to check the optical response of the observed *coral-like* nanopatterns, spectral reflectance measurements have been performed on the produced SERS substrates. This was accomplished by using an UV–Vis spectrometer (Perkin Elmer, Lambda 35) equipped with an integrating sphere for spectral analysis in the 200–1000 nm region. In particular, diffuse reflectance spectra were acquired by setting the sphere reflectance port at 0$^{\circ }$, therefore excluding the specular reflected beam. Figure 3b shows the diffuse reflectance curve obtained for the selected nanopattern. As it is possible to see, it exhibits an edge below 400 nm and a diffuse reflectance in the 15–23% range in the visible and near-IR region. Clearly, the observed broad resonances can be ascribed to the overlapping contributions of either localized surface plasmons associated with single particles (reasonably associated with the resonance centered around 550 nm), or yielded by the complex inter-particles coupling. However, although it is quite difficult to deconvolve the observed spectra from its potential contributions, the obtained broad plasmonic response enables the use of these substrates for multi-wavelengths SERS analysis.

#### Spatial uniformity

**Figure 4:**
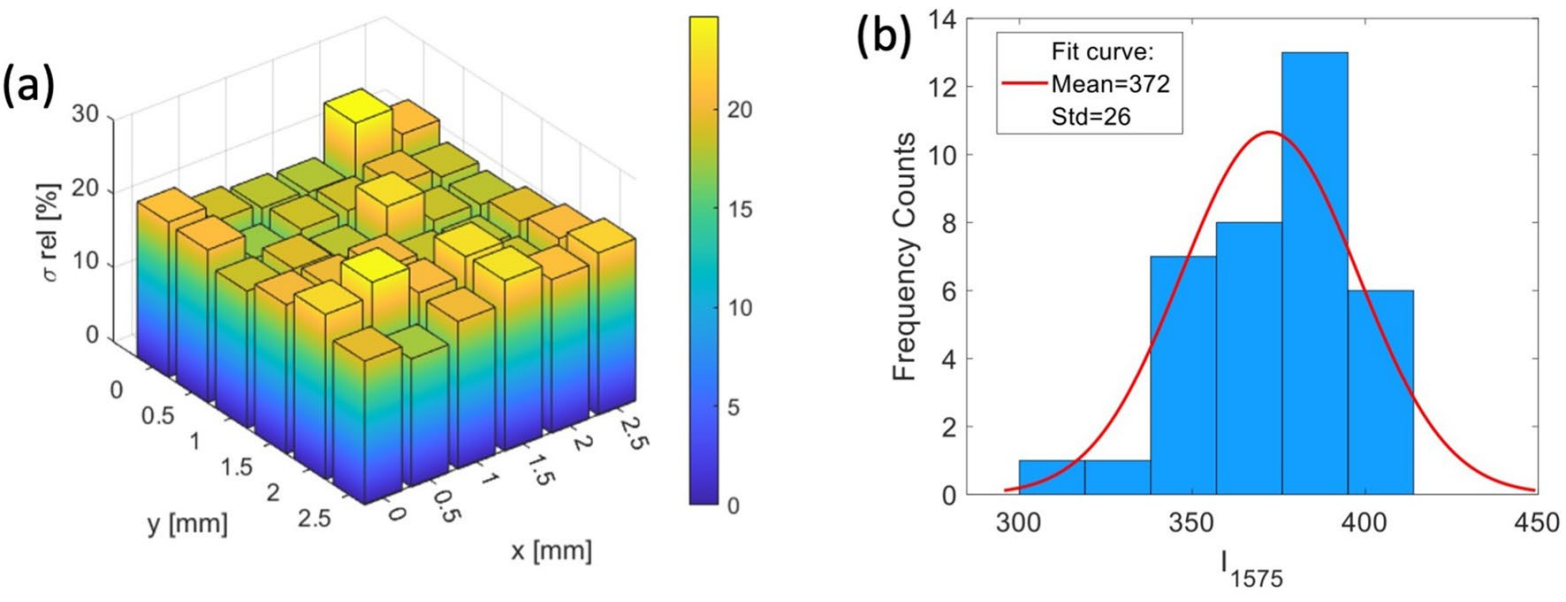
**(a)** Percentage variability $\sigma$/$\langle \hbox {I}_{{1575}}\rangle$ observed in 36.50 $\upmu$m $\times$ 50 $\upmu$m maps uniformly distributed in a 6.25 mm$^2$ square area . **(b)** Distribution of $\langle \hbox {I}_{{1575}}\rangle$ values obtained from 36 maps, together with the data fitting with a Gaussian curve.

As it is well known, the reproducibility of SERS signals acquired within the same substrate is a crucial factor for SERS-based quantitative applications. Thus, this feature was accurately investigated. At this purpose, a 10 mM 4-MBA solution was dried on substrates, according to the procedure previously described. At such concentration, a number of molecules in the confocal area of the order of 10$^{8}$ can be estimated. Therefore, 36 50 $\upmu$m $\times$ 50 $\upmu$m maps, each consisting of a raster scan of 2500 points, were acquired on a 6.25 mm$^2$ square region. In particular, signals were acquired by using a Raman probe power P = 30 $\upmu$W and an integration time $\tau$ = 0.2 s. In these experimental conditions, the spontaneous Raman contribution is negligible. Finally, the intensity of the SERS feature at 1575 cm$^{-1}$, $\hbox {I}_{{1575}}$, has been evaluated in all the acquired spectra, together with the mean value ($\langle \hbox {I}_{{1575}}\rangle$) and the standard deviation $\sigma$. The outcome of this analysis is reported in Fig. 4. In particular, Fig. 4a reports the percentage variability ($\sigma$/$\langle \hbox {I}_{{1575}}\rangle$) observed in each map. As is it possible to see, the signal fluctuactions in each map is around 15$\%$, which represents a very good result considering the wide explored area. Notably, the average signal $\langle I_{1575}\rangle$ obtained in the different maps are consistent each other, indicating a reasonable homogeneity of the substrate plasmonic performances. The $\langle \hbox {I}_{{1575}}\rangle$ values distribution is reported in Fig. 4b, together with a fitting of the histogram with a Gaussian curve. From the fitting parameters it is possible to estimate a relative standard deviation for $\langle \hbox {I}_{{1575}}\rangle$ $\sim$ 7%. Similar results have been obtained for maps randomly distributed on the whole substrate area A (A = 2.25 cm$^2$), despite to the centimeter scale translation.

#### Substrate aging, contamination and wettability

As it is well known, Ag-SERS substrates tend to degrade with time when exposed to water or air^46–48^. Moreover, also carbonaceous species can be adsorbed on the substrate, limiting the SERS sensitivity as interfering contaminants. For this reason, SERS substrates are ideally stored under vacuum conditions, in order to avoid both silver oxidization (mainly occurring in presence of water vapor) and contamination. In the case of our substrates, in order to avoid the necessity of storage under vacuum, it is possible to froze the substrate preparation protocol just after the air-based plasma treatment. In this phase, oxidized substrates can be also stored under environmental condition. Clearly, the final 50 s lasting Ar-plasma treatment can be applied just before the effective substrates use.

**Figure 5:**
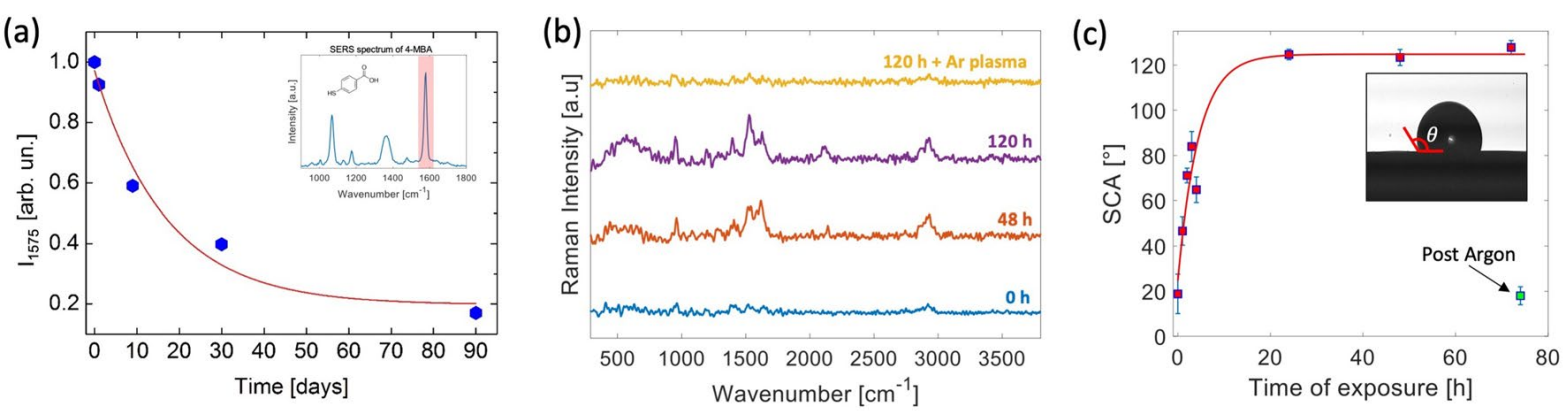
**(a)** SERS signal intensity vs the substrate storage time. The signals were normalized to the value observed at t = 0. The graph also reports a fit of the data with an exponential decay curve. **(b)** SERS signals from an initially clean substrate, following exposure to room environment. The exposure times are indicated by the labels. **(c)** SCA values vs the exposure time to room air. The insets show images of a 2 $\upmu$L drop deposited on the substrate, from which the SCA was obtained.

Said that, we also investigated the aging effect of substrates following the full RF treatment. At this purpose, a 1 $\upmu$M 4-MBA solution was deposited on a freshly prepared substrate, according to the procedure previously described. Therefore, SERS spectra were acquired on 50 points randomly selected on the whole substrate. The same procedure was repeated after different storage intervals of the substrate at room air (humidity $\sim$ 30%, T $\sim$ 24 $^{\circ }$C). Figure 5a reports the $\hbox {I}_{{1575}}$ value, averaged on the acquired points, obtained after different storage intervals, normalized to that corresponding to a freshly prepared sample. As it is possible to note, the average signal undergoes a 40% drop after 10 days, which becomes as high as 80% after 3 months. However, in this condition, the visibility of SERS signal is still high. More tricky is, instead, the effect of substrate contamination, especially when SERS analysis is performed in the so-called single-molecule regime. As a matter of facts, when a clean substrate is exposed to room air, contamination with carbonaceous materials occurs, mainly due to PAH molecules present as air pollutants at trace level ($\sim$ ng/m$^3$). As results, a background signal arises in SERS spectra, mainly in the 1300–1800 cm$^{-1}$ region. This effect is shown in Fig. 5b, which compares the background spectra of a clean substrate (no sample added), and that obtained after different times of exposure to room environment, as indicated in the spectra labels. Also in this case, the spectra result from averaging of 50 randomly selected points on the whole substrate. Clearly, this background strongly impairs the detection of analytes at low concentration, with a degradation of the limit of detection. Intriguingly, such contaminations have also a direct impact on substrate wettability^49^, a parameter which has to be taken into account for quantitative SERS analysis. As a matter of facts, while strongly hydrophobic substrates are able to concentrate on the substrate molecules from strongly diluted solutions, therefore intrinsically improving the intensity of the acquired signal^50^, hydrophilic substrates are instead preferred to obtain a uniform distribution of molecules on the substrate, avoiding spurious effects (e.g. coffee ring effect)^51^. Moreover, hydrophilic substrates should also be preferred for the integration in microfluidic SERS-based detection platforms. In view of this, we also analyzed the substrate wettability and its modification following storage at room environment. In Fig. 5c we report the static contact angle (SCA) vs the exposure time to room air. As it possible to see, the freshly prepared substrate is markedly hydrophilic (SCA $\sim$ 20$^{\circ }$), mainly due to exposure to Ar plasma. Following exposure to air, SCA increases, reaching a plateaux value of $\sim$ 125$^{\circ }$ after $\sim$ 24h. However, we verified that substrates can be efficiently cleaned by contaminants by exposure to the Ar plasma for $\sim$ 60 s, which also fully restores the initial substrate hydrophilicity, with SCA $\sim$18$^{\circ }$.

## Conclusions

In this paper, we propose a rapid, easy, effective and highly reproducible protocol for the fabrication of Ag-based SERS substrates. In particular, we demonstrate that two successive ICP treatments are able to induce a considerable roughening of a flat Ag layer, which exhibits a *coral-like* silver nanopattern. The obtained Ag-nanotexture exhibits a certain level of order as revealed by Fourier Analysis of the morphological features, reasonably resulting from the contribution of spinodal dewetting in the ICP treatment. This surface texture is characterized of a high density of hot-spots, giving rise to a EF up to 6.5 $\times$ 10$^7$ for the non-resonant 4-MBA molecule at 532 nm. The developed approach leads to high sensitive, uniform and low-cost SERS substrates, which can be successfully used in sensing platforms.

## Methods

### Sample preparation

In order to estimate the substrate EF, 4-MBA (Sigma-Aldrich) was used as probe molecule. In particular, the spontaneous Raman spectrum was acquired from a 0.1 M ethanol solution, which was infiltrated into a sample chamber consisting of two sandwiched glass coverslips. For SERS measurements, a 10 $\upmu$L drop of 1 $\upmu$M solution was casted on the substrate and allowed to dry at room temperature.

### Spectra acquisition

Raman and SERS spectra were acquired by using the WiTec Alpha 300 micro-Raman system, endowed with a Raman probe at 532 nm and a 60$\times$ dry objective. Photons back scattered from the sample were allowed to pass through an edge filter and then sent to a spectrometer via a 50-$\upmu$m core fiber, acting as pin-hole for confocal acquisition. In this condition, the confocal area is $\hbox {A}_{{conf}}\sim$ 0.44 $\upmu$m$^2$, while the confocal volume is $V_{conf}\sim$2.3 fL. Raman and SERS spectra from 4-MBA molecules were acquired by using a 15 mW and 10 $\upmu$W laser probe power, respectively.

### Morphological characterization

The morphology of the obtained nanopatterns were examined by a field-emission scanning electron microscope system (SEM), operating at an accelerating voltage of 10 kV. Therefore, the obtained images were analyzed by using ImageJ software. In particular, in order to obtain the characteristic length $\lambda _c$, for each SEM image the 2D FFT $U(\xi _x$, $\xi _y)$ was obtained, and then the Power Spectrum Density (PSD) was calculated according to the relation:

2 $$\begin{aligned} PSD(\xi _x,\xi _y)=\frac{1}{N_x^2 N_y^2} \cdot \frac{|U(\xi _x,\xi _y)|^2}{\Delta \xi _x \Delta \xi _y}, \end{aligned}$$

being $N_{x(y)}$ and $\Delta \xi _{x(y)}$ the number of pixels and the data spacing in each domain, respectively. Finally, the rotational average of PSD was calculated and fitted with a Lorentzian function. From the maximum ($\xi _m$) of this curve, the characteristic length $\lambda _c$ = 1/$\xi _m$ was calculated.

## Supplementary Information

Supplementary Information.
