# Supplementary material for: A simple and reliable approach for the fabrication of nanoporous silver patterns for surface-enhanced Raman spectroscopy applications
Source: Sci Rep. 2021 Nov 16;11:22295. doi: 10.1038/s41598-021-01727-z (PMC8595463; doi:10.1038/s41598-021-01727-z)
Supplement: Supplementary file 1 — Supplementary Information. [file 41598_2021_1727_MOESM1_ESM.pdf]

A simple and reliable approach for the fabrication of nanoporous silver patterns for Surface-Enhanced Raman spectroscopy applications

Angela Capaccio[a], Antonio Sasso[a,b], Giulia Rusciano[a,b,*]

[a]University of Naples Federico II, Department of Physics 'E. Pancini', Via Cintia, I-80126 Naples, Italy
[b]National Institute of Optics (INO)-National Research Council (CNR), Via Campi Flegrei 34, I-80078, Pozzuoli (NA), Italy

*giulia.rusciano@unina.it

Electronic Supplementary Information

1. Morphological analysis of the Ag film sputtered on the Cr/Au adhesion layer.

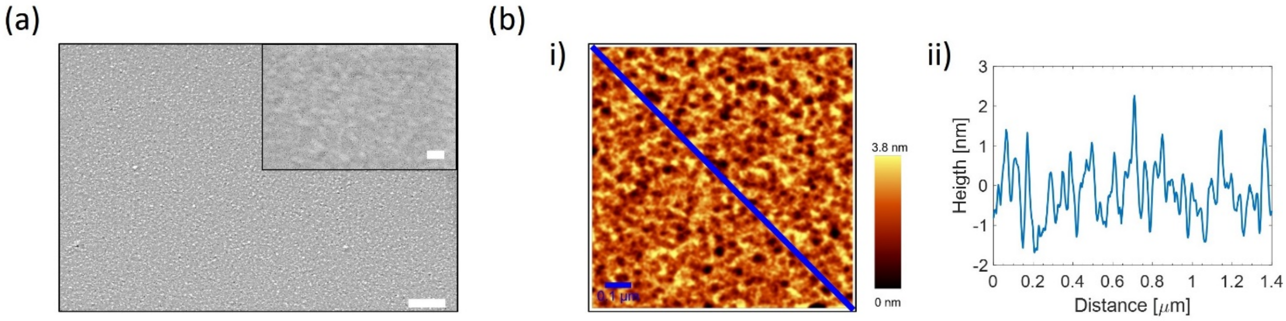

Figure S1: (**a**) SEM images of a 30 nm Ag film sputtered on a Cr/Au adhesion bilayer; (**b**) AFM height map in a 1 μm x 1 μm region (i) and height profile corresponding to the blue line highlighted in the map (ii).

2. Raman analysis of the coral-like nanopattern, formed by application of the air-based plasma.

By irradiating the oxidized nanopattern with a laser power ~ 17 mW, we observed a significant spectra evolution in which the AgO features disappear while two new bands appear, at ~ 803 cm^{-1} and 630 cm^{-1} (Fig. S2a). According to literature [1], both bands can be ascribed to the thermal decomposition of silver oxide to bulk silver. In particular, the band at 803 cm^{-1} is due to an atomic oxygen species (referred to as Oγ) chemisorbed on the reconstructed silver surface. Although O_γ species are confined to the surface, the intensity of this band is relatively high, probably as a consequence of the concomitant SERS effect associated with surface roughness. On the contrary, the band at 630 cm^{-1} is due to oxygen species chemisorbed below the Ag surface, derived from oxygen species diffusion underneath the Ag surface. Such chemisorbed species are referred to as O_β species.

In Fig. S2b we report the time evolution of these bands for the substrate undergoing a continuous irradiation of the Raman laser excitation set at a 17 mW power. As it is possible to note, the band due to O_β species increases, reaching a plateau after ~ 12 s of continuous laser irradiation. At the same time, the band corresponding to O_γ species decreases, reaching a plateau on the same time scale. Reasonably, after 12 s, an equilibrium is reached among O_γ and O_β species. Clearly, the decrease of $O\gamma$ band can be also partly ascribed to the loss of SERS activity of the reconstructed metallic silver surface, due to the continuous diffusion of oxygen species below the surface.

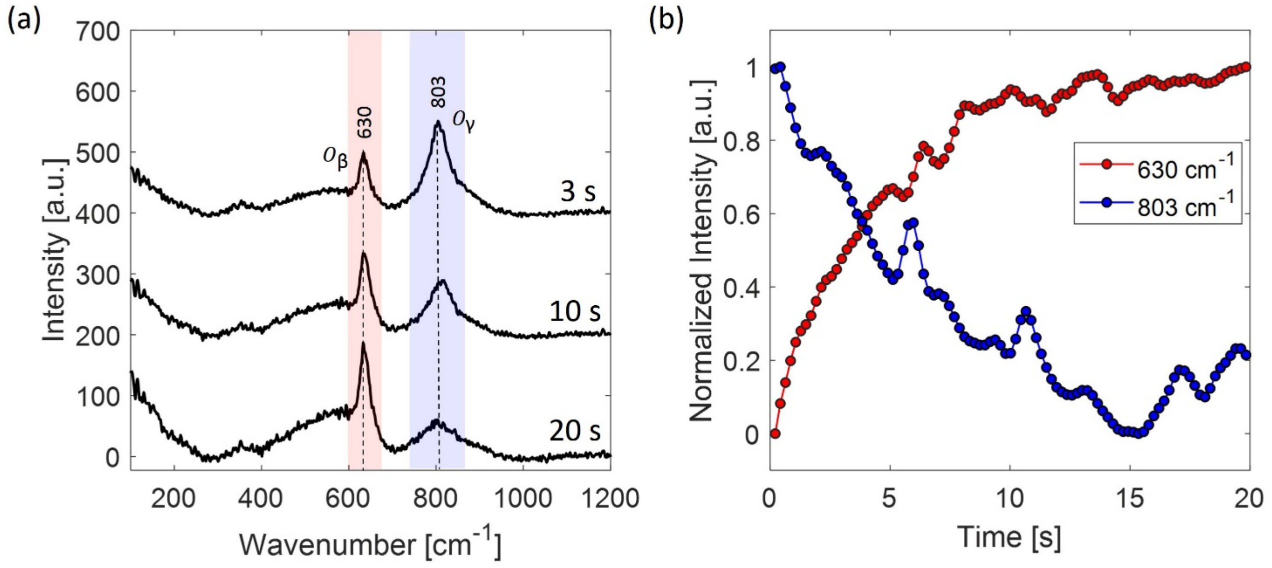

Figure S2: (**a**) Raman spectra recorded on the oxidized substrate during the laser irradiation and extracted at three different times, as indicated in the labels; (**b**) Time evolution of Raman bands highlighted in part (a) corresponding to O_β (pink bar) and O_γ (violet bar) species.

3. EF evaluation by using Adenine as molecular probe.

As it is well known, the measured EF can strongly depend on the selected molecular probe. In fact, even neglecting effects related to chemical enhancement and/or the presence of resonances (which are ruled by the specific electronic and vibrational probe structure), surface affinity of the probe molecule with the SERS surface also plays a major role. Specifically, an analyte with a high binding affinity with the surface will "feel" more effectively the plasmonic enhancement with respect to a poor binding analyte. As result, a different EF will be experimentally found in the two cases. For this reason, the choice of the analytical probe for EF evaluation has to be carefully evaluated. According to ref. [2], adenine (as 4-MBA), can be a reliable and effective molecular probe. This molecular species presents a high affinity to silver or gold and is non-resonant for excitation at 532 nm. Therefore, in this investigation, EF was also estimated by using this molecular specie.
At this purpose, a 'mother solution' was prepared by dissolving adenine molecules (from Sigma-Aldrich) in distilled water (pH=7) at a 0.1 M concentration. Hence, a 1 µM solution was prepared through successive dilutions and spread on the SERS substrate according to the procedure previously described for 4-MBA molecules. Figure S3 compares the spontaneous Raman (lower trace) and the SERS (upper trace) spectrum obtained in this investigation. In particular, the spontaneous Raman spectrum was acquired by using a Raman probe power P_R=27 mW and an integration time τ_R=200 s, while a Raman probe power P_S=30 µW and an integration time τ_S=2 s were used for the SERS case. The EF was finally estimated by using the adenine ring breathing mode around 730 cm^{-1}, highlighted in figure S3 by the pink bar. In

these conditions, we obtained EF~8·10^6. It is worth noticing that for this molecular species a strong dependence of the EF can be observed from the pH value of the adenine buffer solution [3]. In particular, changing the pH value from 7 to 9, the measured EF increases of almost an order of magnitude.

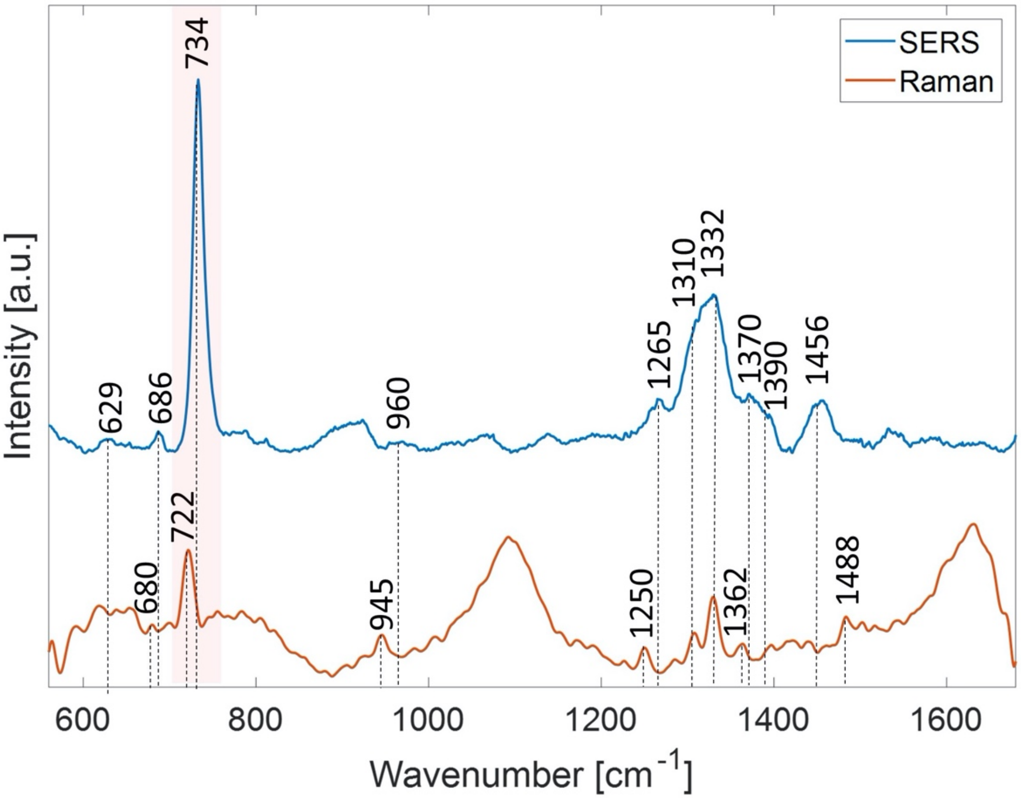

Figure S3: comparison between the Raman spectrum of 0.1 M adenine solution (lower trace) and the SERS spectrum of a 10^{-6} M adenine solution adsorbed on the SERS substrate (upper trace).

[1] B. Pettinger, X. Bao, I. Wilcock, M. Muhler, R. Schlgl, G. Ertl, "Thermal decomposition of silver oxide monitored by raman spectroscopy: From AgO units to oxygen atoms chemisorbed on the silver surface", Angew. Chem., Int. Ed. Engl. 33 (1), 85-86, (1994).

[2] S. E. J. Bell, G. Charron, E. Cortés, J. Kneipp, M. Lamy de la Chapelle, J. Langer, M. Procházka, V. Tran, S. Schlücker, "Towards Reliable and Quantitative Surface-Enhanced Raman Scattering (SERS): From Key Parameters to Good Analytical Practice" - Angew. Chem. Int. Ed. 59, 5454-5462, (2020).

[3] Y. Tzeng, B. Lin, "Silver-Based SERS Pico-Molar Adenine Sensor" Biosensors 10, 122, (2020).
